# The Institutional Library

**Published:** 1920-11-20

**Authors:** 


### THE INSTITUTIONAL LIBRARY.

**Adenoids and Enlarged Tonsils Curable without Operation.** By John Kynaston, Lt.-Col. R.A.M.C. (retired). (London: St. Catherine Press. 1s. net.)

That a certain number of unnecessary operations for tonsils and adenoids are performed by the less discriminating of the medical profession, few will be found to deny; and it is equally common ground that in a certain percentage of cases where even the best instructed might advise operation, improvement may take place spontaneously or during any course of remedial treatment. But that the vast bulk of cases of "tonsils and adenoids" are thus amenable to medicinal or physical treatment Colonel Kynaston offers us no shadow of proof whatever. His pamphlet we regard as little short of a misfortune to any parent or guardian into whose hands it may fall. He draws lurid and quite misleading pictures of the dangers of the operation, glosses over the immense benefits which it confers in properly selected cases, and ends with dogmatic promises of "cure" by certain simple measures long tested and perfectly well understood by the medical profession. He does not emphasise the numerous and serious sequelæ of neglected tonsils and adenoids—middle-ear disease and its complications, for example—and he conveys to the public (for whom the booklet appears to be written) an impression which we believe to be both untrue and unsupported by the very scanty evidence he adduces for it. We do not question his *bona fides*, but only his competence to constitute himself a mentor of parents on this particular question; and the general tone of the pamphlet is equally disappointing.

**Mechanical Dentistry:** A Practical Treatise on the Construction of the Various Kinds of Artificial Dentures. By Charles Hunter. Seventh Edition. (London: Crosby Lockwood & Son.)

This book is introduced to the dental profession from an essentially practical standpoint. As a text-book it is already well known, and in the latest edition it keeps thoroughly abreast of the progress of the times. A considerable number of new illustrations has been added, and much of the matter has been rearranged and rewritten.

**Early History of Surgery in Great Britain.** By G. Parker, M.A., M.D., M.R.C.S. Illustrated. (London: A. & C. Black, Ltd. Price 7s. 6d. net.)

This small volume forms part of the Medical History Manuals of which Dr. J. D. Comrie is general editor. In a highly interesting manner are traced the revival and early surroundings of the surgical art after the end of the dark ages of Europe. The author has taken considerable pains to investigate many questions connected with the ancient therapeutic guilds in this country. He also has a happy knack of including the social and political influences in connection with the professional thought of the time, without in any way losing sight of the original object of his historical sketch—namely, surgery as such. We heartily recommend his work as both interesting and highly instructive reading. The illustrations are excellent.

**The Critical Age of Women.** By Walter M. Gallichan. (T. Werner Laurie. 1920. Pp. 160. Price 6s. net.)

This book is not solely concerned, as its title might suggest, with the menopause; it roves discursively also over many topics connected with the sexual functions of woman, from the cradle to the grave. As a practical guide to conduct and hygiene during the menopause, it is far inferior to Dame Mary Scharlieb's recently-published book on this subject, but it does contain much that is interesting and useful for women to know about their natural sex functions. It is written with candour, and with an excellent moral tone.

**Personal Hygiene.** By M. R. Samey, M.D. (Calcutta: Butterworth & Co. 3 Rs.)

With the best of intentions, the author has followed a method in writing this book which leads nowhere and helps no one. That method is the stringing together of controversial quotations from quite unknown or undistinguished authorities. It is not easy to guess whether the book is addressed primarily to the British in India or to the Indians; while there is much in it that might be useful to both, there is also much that is nonsensical, and advantageous to neither. The author's description of his own work as "the lame labours of this limping Lilliput" is too humorous to be passed over in silence.

**The Education Act, 1918.** By Arthur A. Thomas, M.B.E., B.A. (London: P. S. King & Son, Ltd. Price 6s. net.)

The provisions of the Education Act and the many points in which it will conflict with the existing habits of citizens are very little understood by the public. Mr. Thomas's manual is intended for the use of administrators, members of local education authorities, school managers, and others interested in education, as well as for the legal profession. It gives the complete text of the Act, and explains concisely the bearing of each paragraph. Ordinary folk will certainly be puzzled when they find they can no longer have a little boy to clean boots and knives before going to school, and must spare their young maids for instruction in the daytime.

